# Supplementary material for: The generation and use of recombinant extracellular vesicles as biological reference material
Source: Nat Commun. 2019 Jul 23;10:3288. doi: 10.1038/s41467-019-11182-0 (PMC6650486; doi:10.1038/s41467-019-11182-0)
Supplement: Supplementary file 1 — Supplementary Information [file 41467_2019_11182_MOESM1_ESM.pdf]

The generation and use of recombinant extracellular vesicles as biological reference material

Geeurickx et al.

Supplementary information

Supplementary figure 1

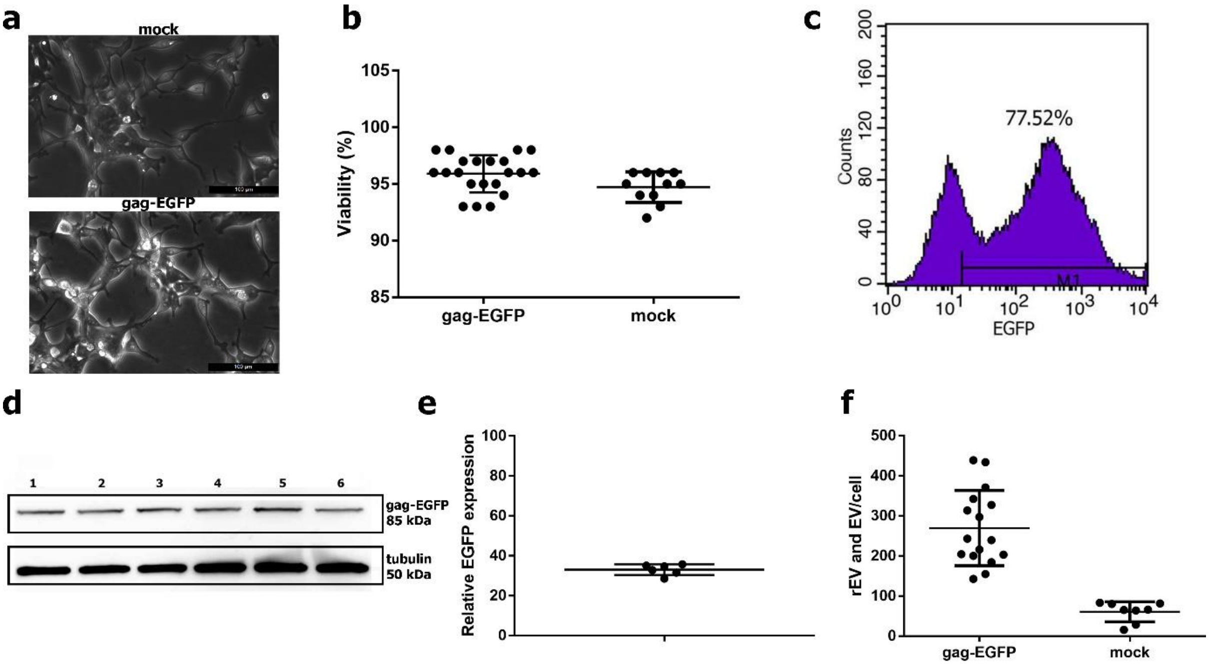

Supplementary figure 1: Validation of the cell culture model used for rEV production. (a) Phase contrast microscopy images and (b) cell viability of HEK293T cells transfected with gag-EGFP and mock DNA (n>11 biological replicates). (c) Transfection efficiency measured by flow cytometry of HEK293T cells transfected with gag-EGFP DNA gated for EGFP (image representative of 3 biological replicates). (d) Western blot analysis for EGFP and loading control tubulin of lysates from gag-EGFP transfected HEK293T cells (n= 6 biological replicates) (20 µg of protein was loaded). (e) Transfection stability assessed by calculation of the relative EGFP expression evaluated by western blot presented in (d). (f) Comparison of the number of rEV and EV per cell produced by gag-EGFP transfected versus mock transfected HEK293T cells quantified by NTA (n>8). Data in b, e, f are (mean, SD). Source data are provided as a source data file.

Supplementary figure 2

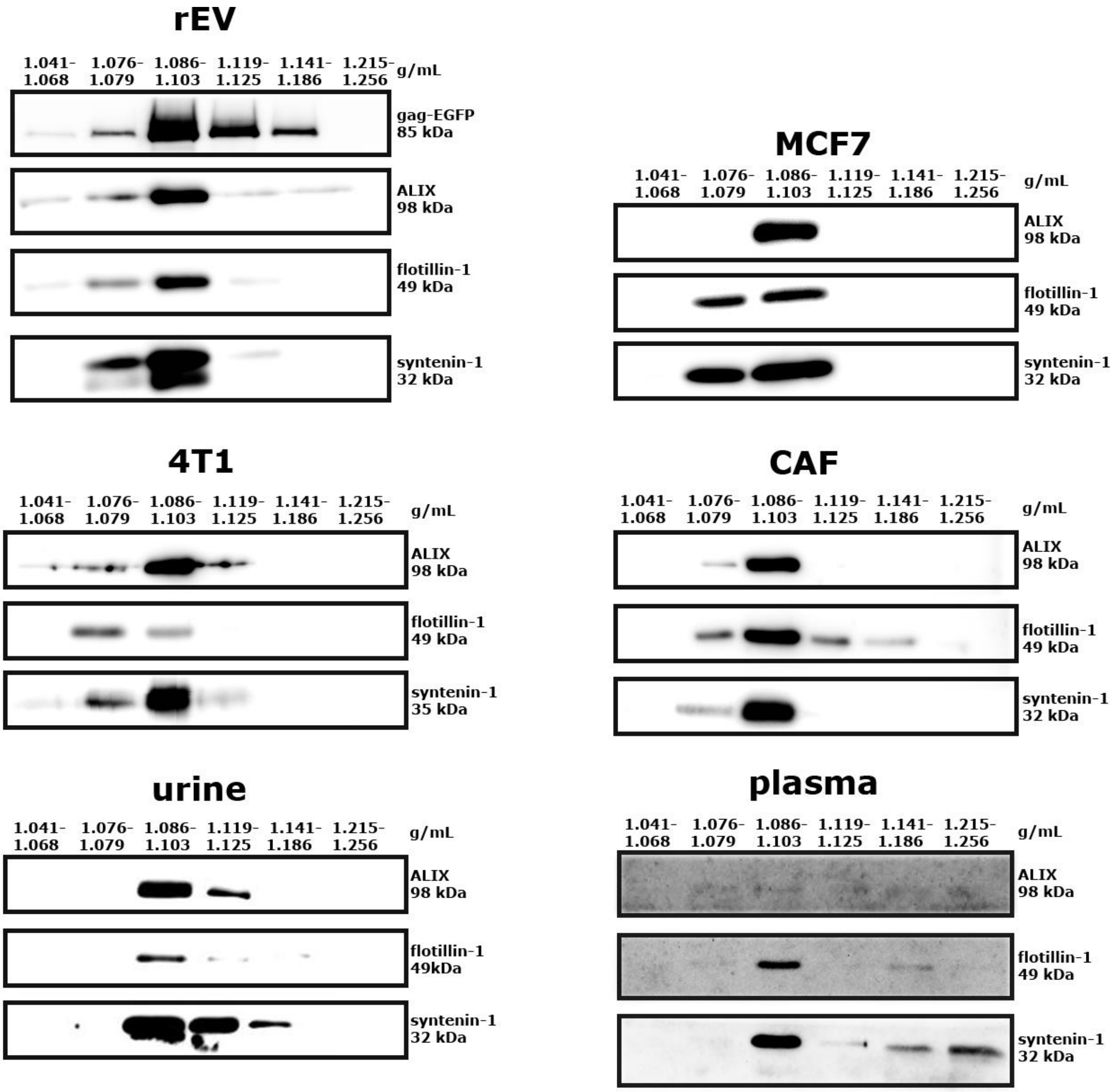

Supplementary figure 2: rEV and sample EV have equivalent buoyant densities. Density distributions of rEV separated by ODG centrifugation of medium conditioned by gag-EGFP transfected HEK293T cells and sample EV separated by ODG centrifugation from medium conditioned by breast cancer cells (MCF7, 4T1) or cancer associated fibroblasts (CAF) or plasma and urine as analyzed by western blot for EGFP and EV-associated proteins ALIX, flotillin-1 and syntenin-1 (all density fractions were loaded in equal volumes) (image representative of at least three biological replicates). Source data are provided as a source data file.

Supplementary figure 3

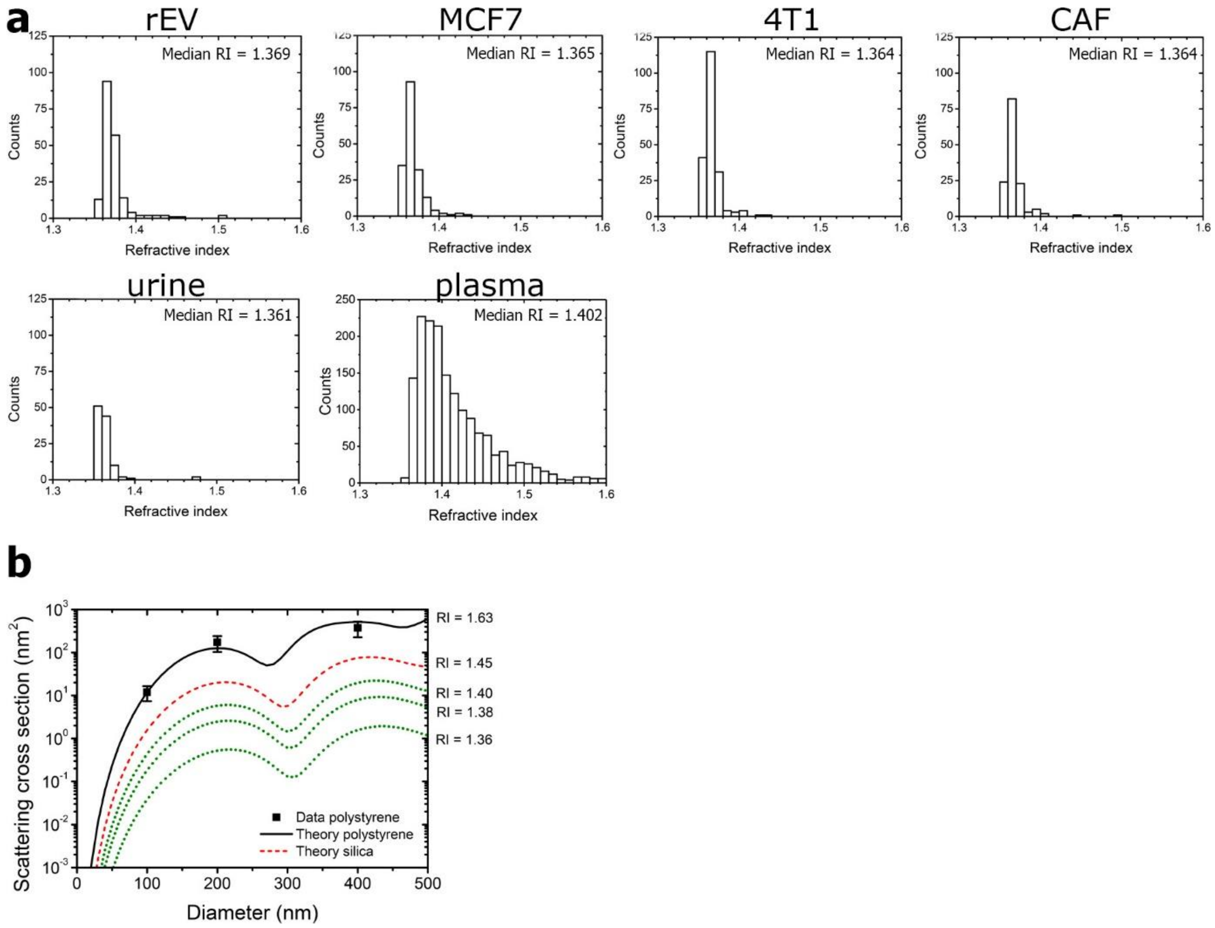

Supplementary figure 3: Refractive index (RI) calculation of rEV and sample EV. (a) RI distributions of rEV separated by ODG centrifugation from medium conditioned by gag-EGFP transfected HEK293T cells and sample EV separated by ODG centrifugation from medium conditioned by breast cancer cells (MCF7, 4T1) or cancer associated fibroblasts (CAF) or plasma and urine calculated by NTA and Mie theory. (b) Measured (symbols) versus Mie theory-based calculations (lines) of scattering cross section versus diameter for polystyrene (black) and silica beads (red) and particles with an RI of 1.36, 1.38 and 1.40.

Supplementary figure 4

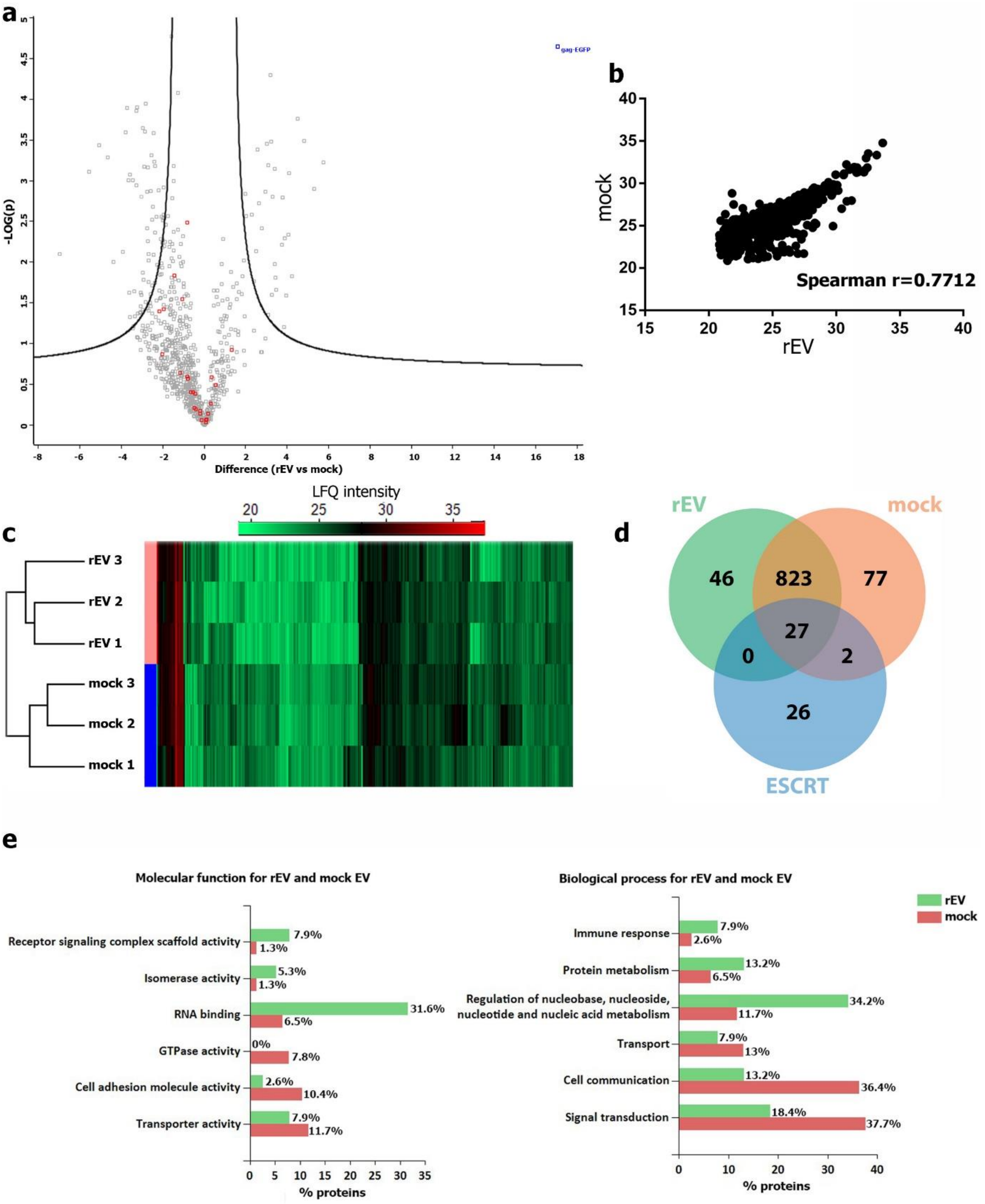

Supplementary figure 4: Proteome comparison of rEV and mock EV. Equal numbers of rEV and mock EV separated by ODG centrifugation from medium conditioned by respectively gag-EGFP transfected and mock transfected HEK293T cells, were subjected to LC-MS/MS. (a) Volcano plot of the fold changes of all detected proteins with a FDR of 0.01 in mock EV (left) and rEV (right). EV-associated proteins are indicated as red dots and the gag-EGFP fusion protein is uniquely detected in rEV (blue dot) (n= 3). (b) Spearman correlation plot of LFQ intensities of all proteins with a FDR of 0.01 from rEV and mock EV, excluding the gag-EGFP protein. (c) Corresponding heat map of all proteins with a FDR

of 0.01 detected in rEV and mock EV. (d) Venn diagram of proteins from the ESCRT pathway, and proteins detected in rEV and mock EV. (e) Comparison of the top 6 molecular functions and biological processes attributed to the proteins enriched in rEV and mock EV. Source data can be accessed at the PRIDE database (PXD010269).

Supplementary figure 5

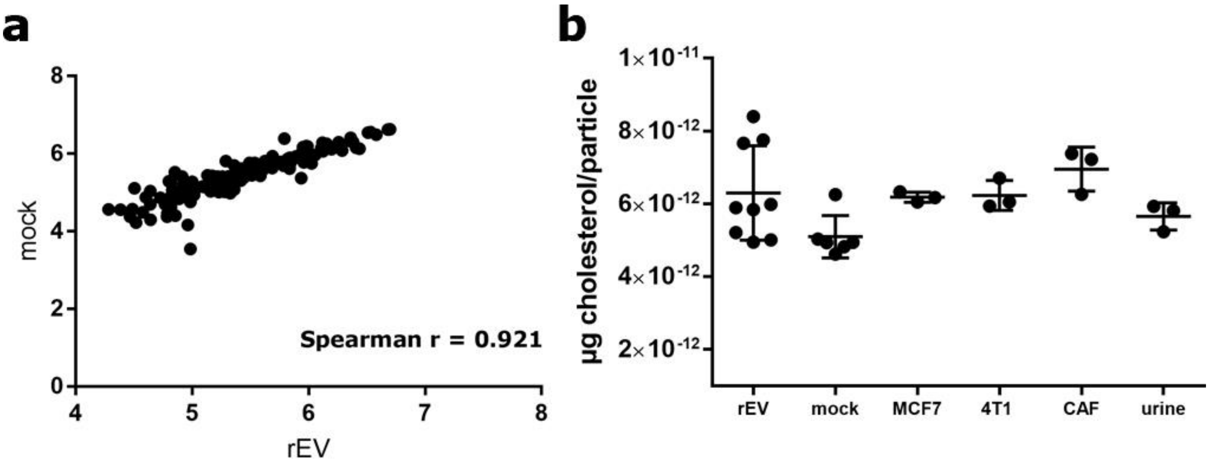

Supplementary figure 5: Lipidome comparison of rEV and mock EV. Lipids were extracted from equal numbers of rEV and mock EV separated by ODG centrifugation from medium conditioned by respectively gag-EGFP transfected and mock transfected HEK293T cells and were subjected to ESI-MS/MS. (a) Log of relative expression values of all detected phospholipids in rEV and mock EV (n=3). (b) µg cholesterol per particle of rEV and mock EV separated by ODG centrifugation from medium conditioned by respectively gag-EGFP transfected and mock transfected HEK293T cells and sample EV separated by ODG centrifugation from medium conditioned by breast cancer cells (MCF7, 4T1) or cancer associated fibroblasts (CAF) or urine. (n=9 (rEV), n=6 (mock EV), n=3 (sample EV from MCF7, 4T1, CAF and urine)) data are presented as (mean, SD). Source data are provided as a source data file.

Supplementary figure 6

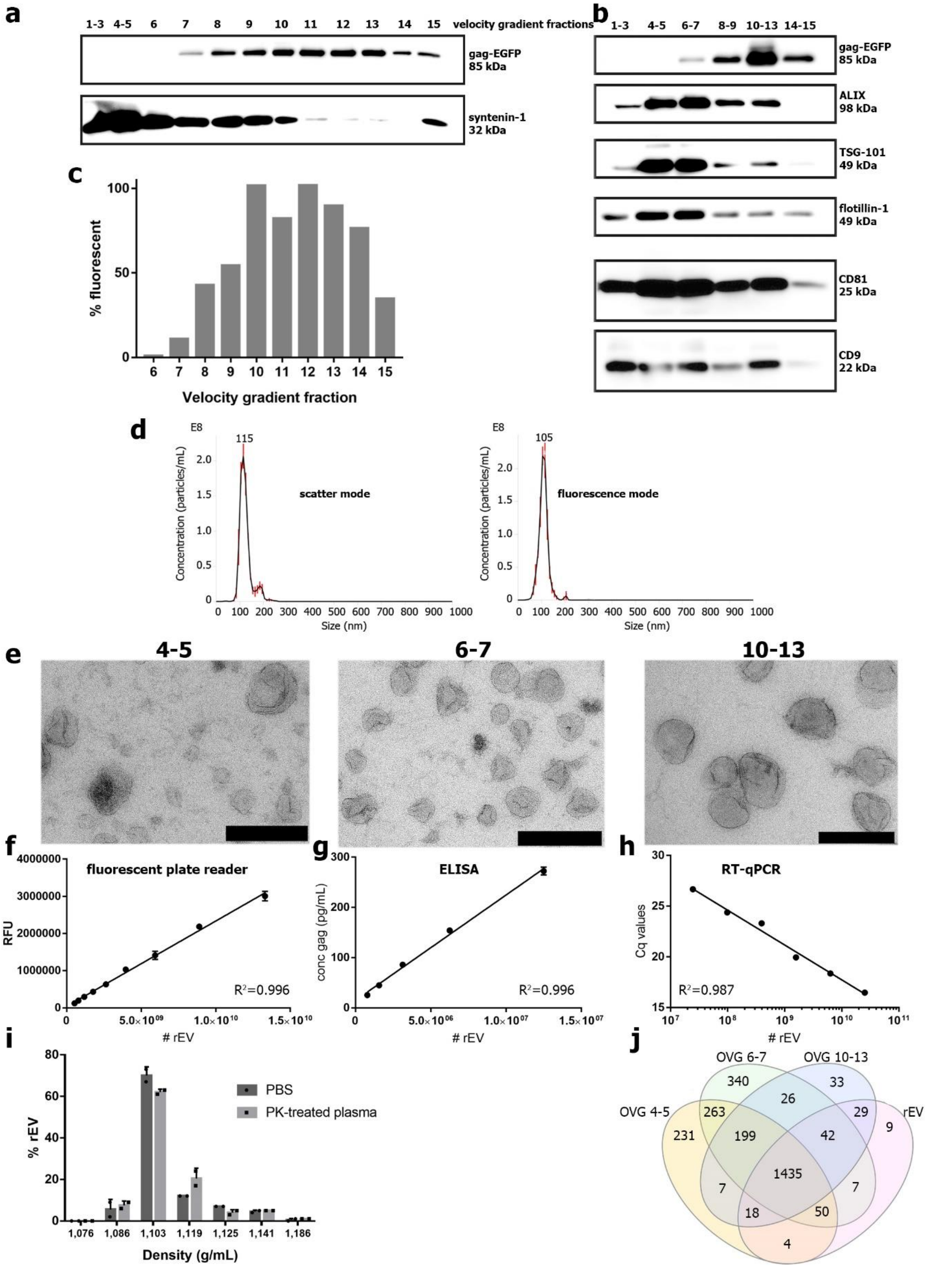

Supplementary figure 6: rEV separation by optiprep velocity gradient (OVG) centrifugation. Western blot analysis for gag-EGFP and EV-associated proteins ALIX, TSG101, flotillin-1, syntenin-1, CD81 and CD9 of (a) individual OVG fractions collected from top (1) to bottom (15) or (b) pooled OVG fractions from top (1-3) to bottom (14-15) obtained after OVG centrifugation of medium conditioned by gag-

EGFP transfected HEK293T cells. (c) Percentage of fluorescent rEV (ratio fNTA/NTA) in individual OVG fractions and (d) size distributions of OVG separated rEV measured with NTA under both scatter and fluorescence mode. (e) TEM images of EV (OVG fractions 4-5, 6-7) and rEV (OVG fractions 10-13) obtained by OVG centrifugation of medium conditioned by gag-EGFP transfected HEK293T cells. Indirect quantification of rEV separated by OVG centrifugation from medium conditioned by gag-EGFP transfected HEK293T cells using (f) a fluorescent plate reader showing a linear correlation of the relative fluorescence units (RFU) and number of rEV, (g) an ELISA for p24 showing a linear correlation of p24 concentration and number of rEV and (h) RT-qPCR for EGFP mRNA showing a semi-logarithmic correlation with the Cq values and number of rEV. (i) Density distribution of OVG separated rEV spiked in PBS and proteinase K (PK)-treated plasma obtained by ODG centrifugation followed by fNTA measurement of ODG fractions (n=2). Data are presented as (mean, SD) (j) Proteomic comparison of endogenous EV (OVG fractions 4-5 and 6-7) and rEV (OVG fraction 10-13) separated by OVG centrifugation versus rEV separated by ODG centrifugation from medium conditioned by gag-EGFP transfected HEK293T. Source data are provided as a source data file.

Supplementary figure 7

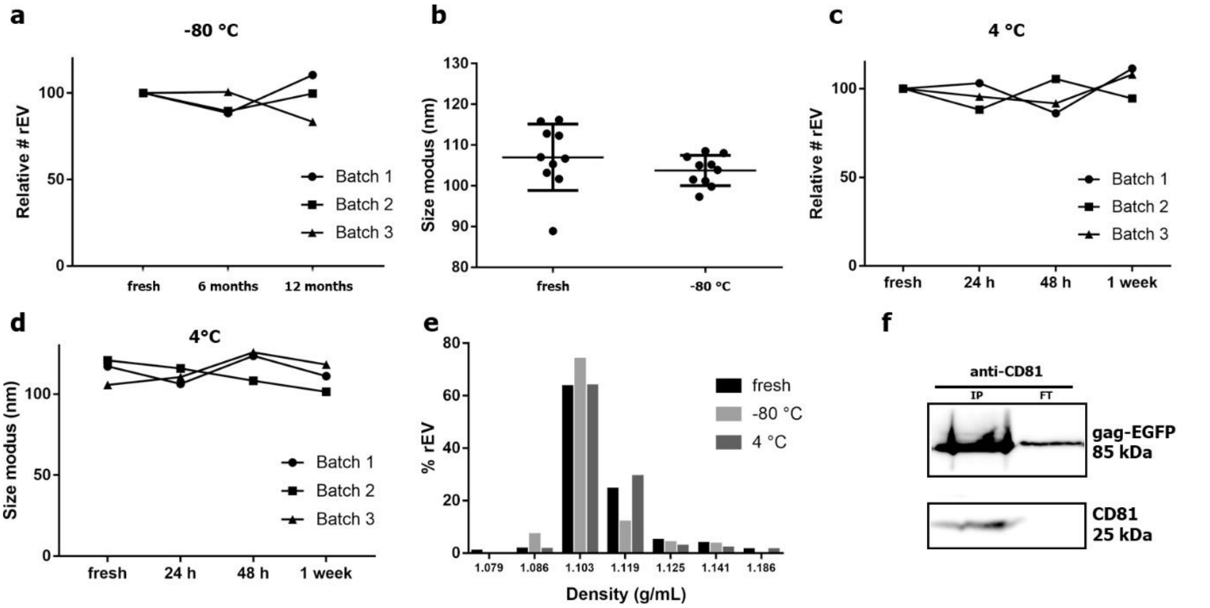

Supplementary figure 7: Stability of rEV at -80 °C or 4 °C. The structural and biological stability of rEV was assessed in different storage conditions. (a) Relative rEV concentration and (b) size modus measured by fNTA immediately upon rEV separation by ODG centrifugation of medium conditioned by gag-EGFP transfected HEK293T cells (fresh) and after 6 months -80°C freeze followed by thaw or 12 months -80°C freeze followed by thaw. Data are presented as (mean, SD). (c) Relative rEV concentration and (d) size modus measured by fNTA of fresh rEV or after storage at 4 °C up to one week (n=3). (e) Density distribution of differently stored rEV (fresh, -80 °C or 4 °C) spiked in PBS obtained by ODG centrifugation followed by fNTA measurement of density fractions. (f) Western blot analysis for gag-EGFP and CD81 of -80 °C stored rEV spiked in PBS followed by immune precipitation (IP) with anti-CD81 coated magnetic beads together with the flow through (FT). Source data are provided as a source data file.

Supplementary figure 8

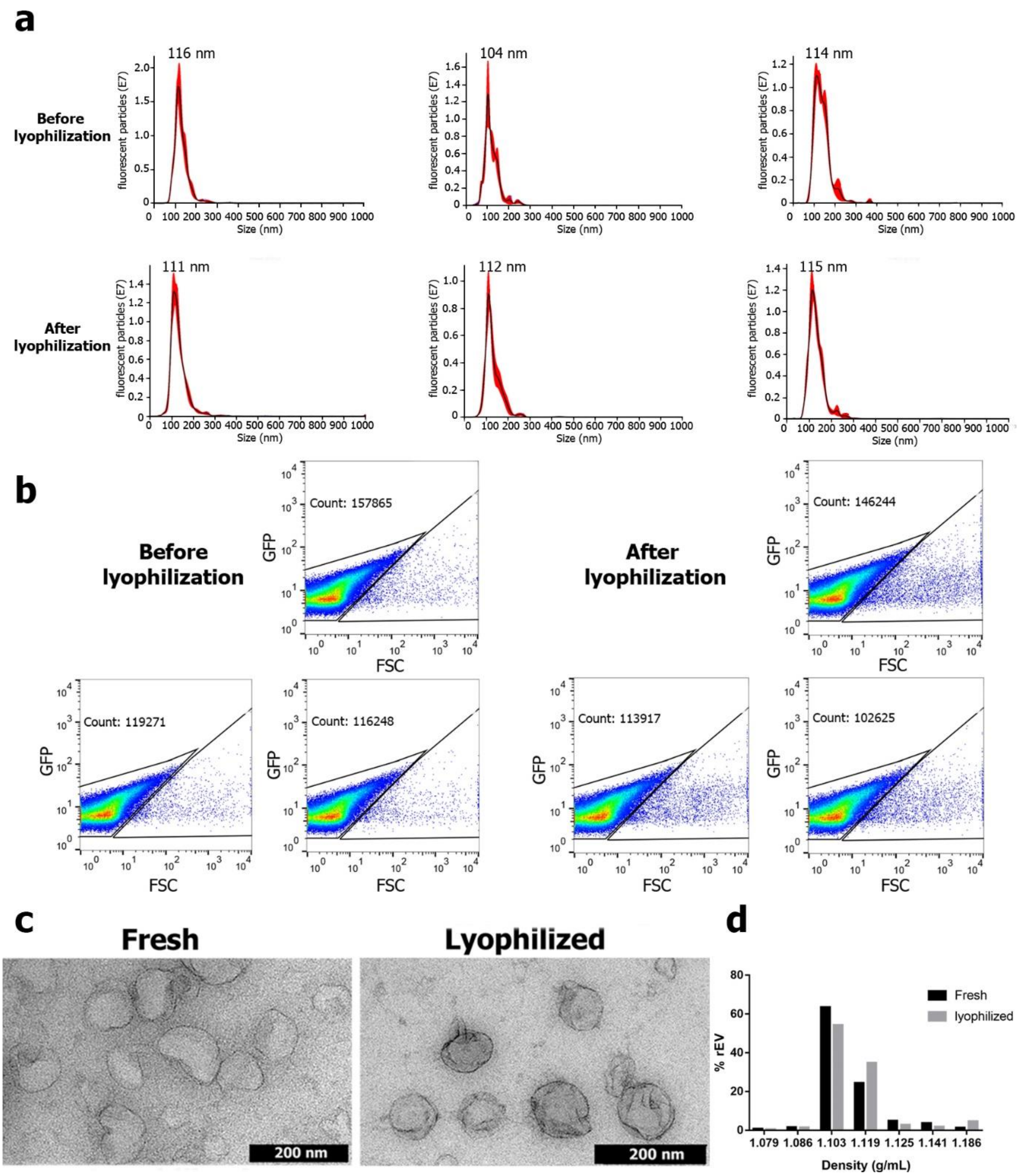

Supplementary figure 8: Lyophilization of rEV. rEV can be lyophilized in PBS supplemented with 5% trehalose without structural and biological changes. (a) Size distribution measured by fNTA of rEV before and after lyophilization (n=3). (b) Scatter plots showing detection of rEV with HR-FC before and after lyophilization (n=3). (c) TEM of rEV before (fresh) and after lyophilization. (d) Density distribution of rEV before (fresh) and after lyophilization in proteinase K (PK) treated plasma obtained by ODG centrifugation followed by fNTA measurement of ODG fractions. Source data are provided as a source data file.

Supplementary figure 9

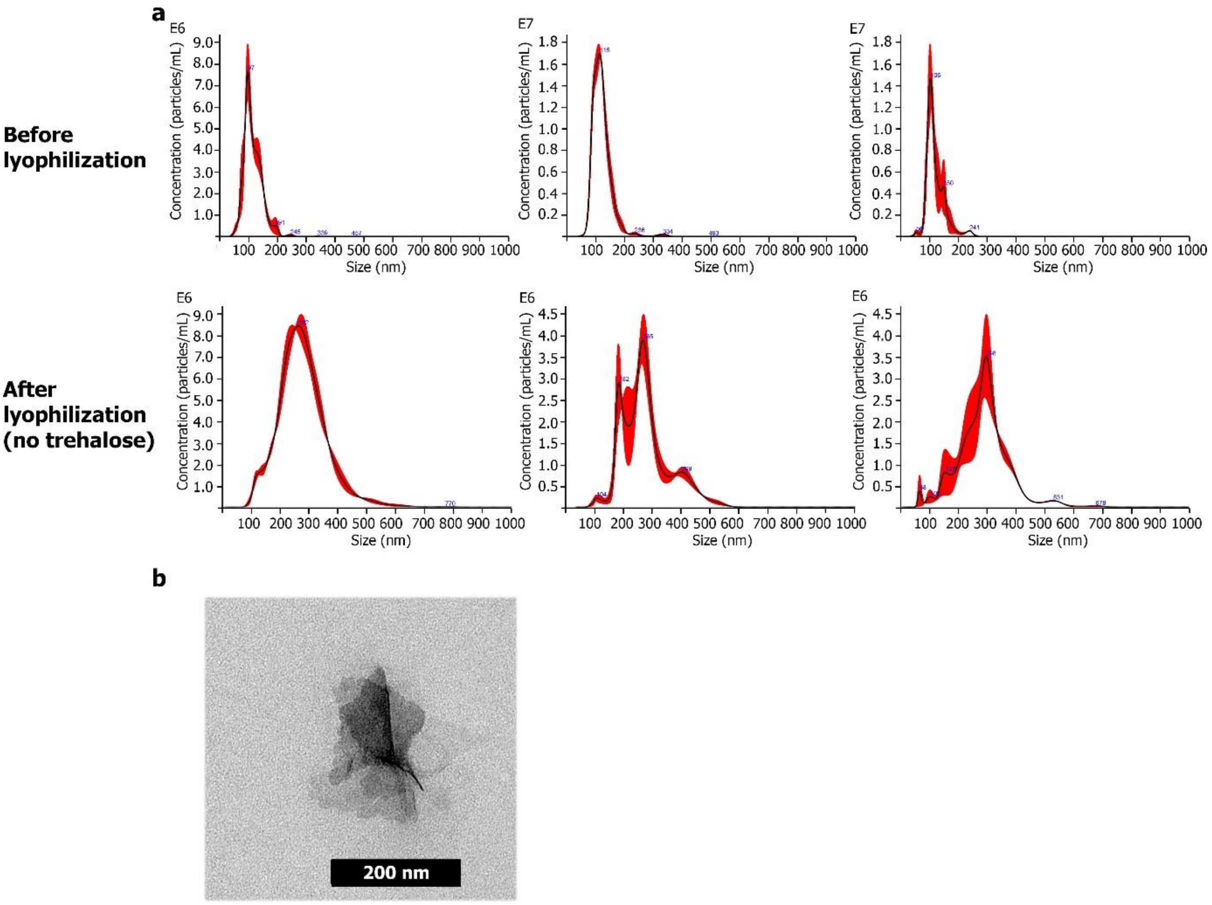

Supplementary figure 9: Lyophilization of rEV without trehalose results in disruption of rEV. (a) Size distribution measured by NTA of rEV before and after lyophilization without addition of trehalose (n=3). (b) TEM of rEV after lyophilization without trehalose. Source data are provided as a source data file.

Supplementary figure 10

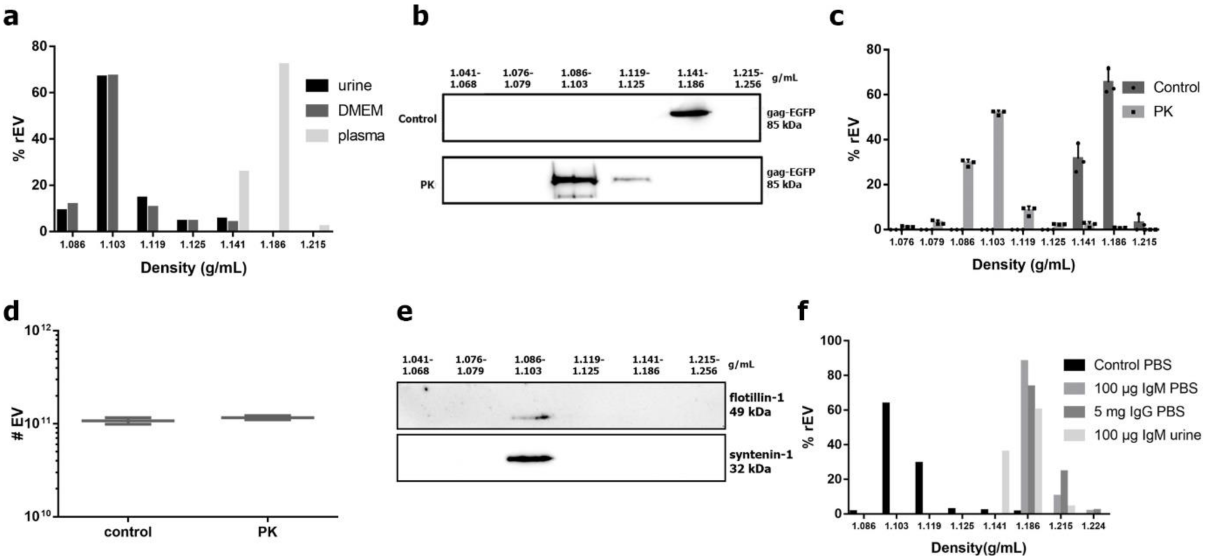

Supplementary figure 10: rEV shift to higher densities in plasma and in the presence of IgM and IgG. (a) Density distribution of rEV spiked in urine, plasma and DMEM supplemented with 10% FBS, obtained by ODG centrifugation followed by fNTA measurement of ODG fractions. (b-c) Density distribution of rEV spiked in proteinase K (PK) treated and control plasma analyzed by (b) western blot analysis for EGFP (image representative of two biological replicates) and (c) fNTA (n=3). (d) Total number of EV in PK treated and control plasma measured by NTA (n=2). (e) Density distribution of EV-associated proteins flotillin-1 and syntenin-1 in PK treated plasma analyzed by Western blot analysis (image representative of two biological replicates). (f) fNTA measurement of ODG fractions reveals rEV density distribution from rEV spiked PBS and urine supplemented or not with IgM/IgG.

Supplementary figure 11

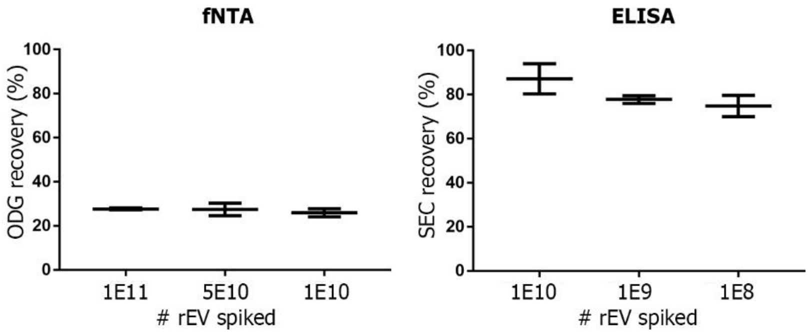

Supplementary figure 11: Recovery efficiency calculation is independent from number of rEV spiked. rEV was spiked in plasma in varying numbers and separated by ODG centrifugation or SEC followed by recovery efficiency calculation with fNTA and ELISA respectively (n=2). Data is presented as (mean, SD). Source data are provided as a source data file.

Supplementary figure 12

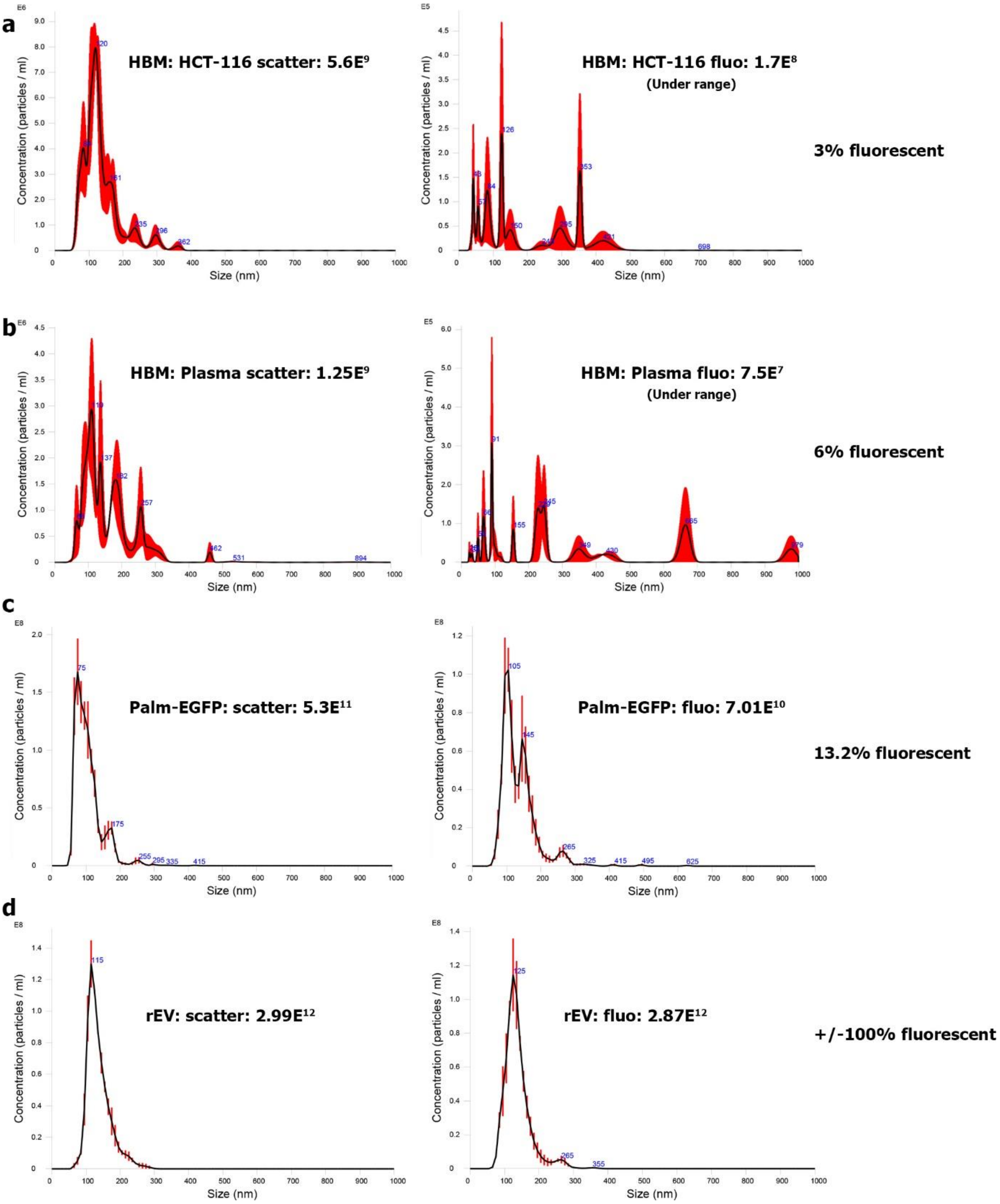

Supplementary figure 12: Comparison of rEV to other potential reference EV. (a) and (b) Size distributions and total number of EV quantified in 1 vial of lipophilic fluorophore stained EV (Hansabiomed life science, HBM) originating from respectively an HCT-116 cell culture model and plasma. Fluorescence measurements resulted in concentrations below the limit of detection ($1x10^8$ particles/mL). (c) Size distributions and total number of EV quantified, separated by ODG centrifugation from medium conditioned by Lck-EGFP transfected HEK293T cells. Lck-EGFP is a membrane-targeted form of EGFP, achieved by fusion of a palmitoylation domain N-terminal of EGFP (Addgene #61099). (d) Size distributions and total number of rEV quantified separated by OVG

centrifugation from medium conditioned by gag-EGFP transfected HEK293T cells. All samples were measured with NTA under both scatter and fluorescence mode with the same settings.

Supplementary figure 13

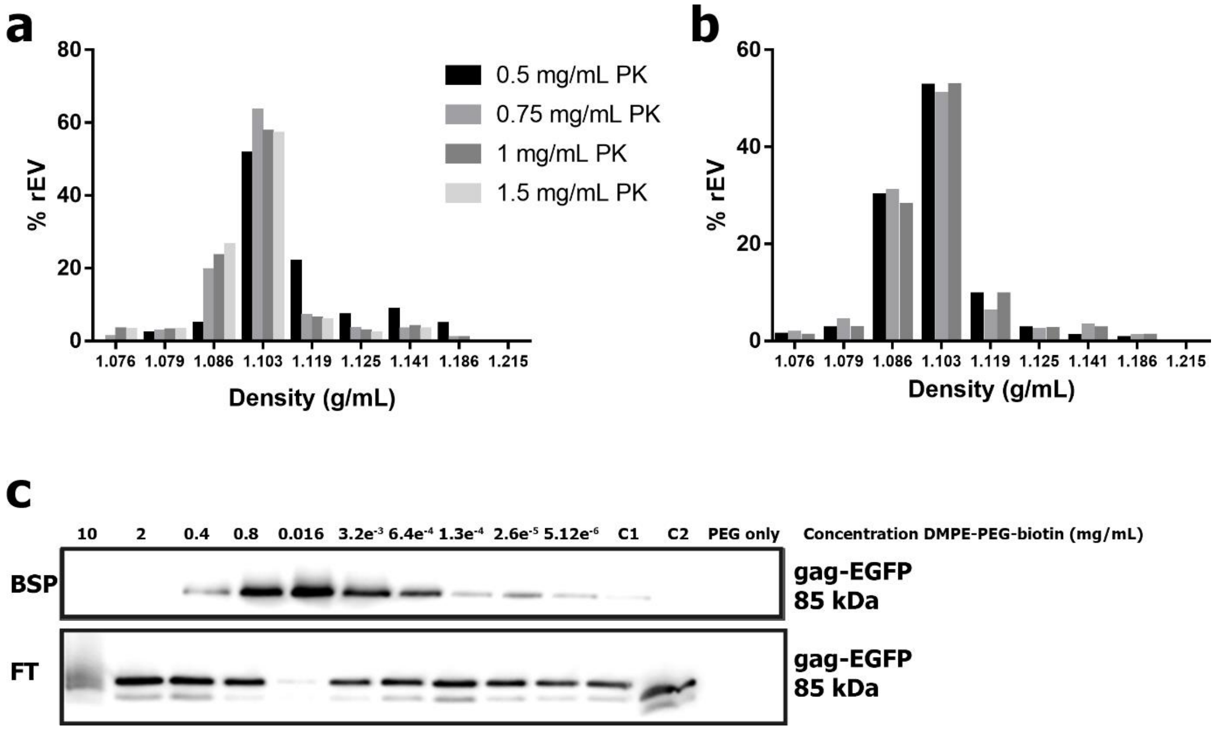

Supplementary figure 13: Optimization of proteinase K (PK) and DMPE-PEG concentrations. (a) fNTA measurement of ODG fractions reveals rEV density distribution of rEV spiked in plasma treated with multiple concentrations of PK and (b) treated with 1 mg/mL PK. (c) Western blot analysis for EGFP of the biotin-streptavidin precipitate (BSP) and flow through (FT) of rEV incubated with different concentrations DMPE-PEG-biotin using streptavidin coated magnetic beads. C1= rEV without DMPE-PEG-biotin, C2= rEV incubated with 0.1 mg/mL DMPE-PEG-biotin on ice, PEG only= the DMPE-PEG-biotin construct without rEV. Source data are provided as a source data file.

Supplementary figure 14

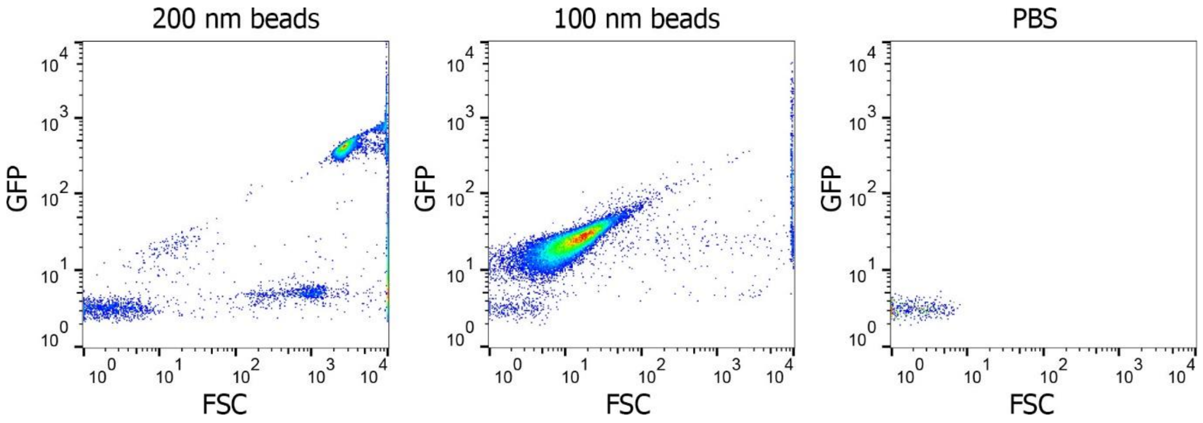

Supplementary figure 14: High resolution flow cytometry (HR-FC) gating strategy: Scatterplots of 200 and 100 nm fluorosphere beads and PBS only for HR-FC.

Supplementary table 1

% sample replaced by PBS	Fluo/mL	Total fluo	Scatter/mL	Total scatter	Efficiency	Normalized scatter	Coefficient of variation (Absolute)	Coefficient of variation (Normalized)
25%	3.75E+08	1.50E+09	5.70E+09	2.28E+10	15.58%	1.46E+11	17.47%	5.94%
12.50%	4.44E+08	1.78E+09	6.93E+09	2.77E+10	18.44%	1.50E+11		
0%	5.45E+08	2.18E+09	8.86E+09	3.54E+10	22.64%	1.57E+11		
25%	3.45E+08	1.38E+09	5.98E+09	2.39E+10	14.33%	1.67E+11		
12.50%	4.65E+08	1.86E+09	7.13E+09	2.85E+10	19.31%	1.48E+11		
0%	5.66E+08	2.26E+09	8.33E+09	3.33E+10	23.51%	1.42E+11		

Supplementary table 1: rEV mitigate for inter-user variability. EV separation was performed in 6 technical replicates from PK treated plasma spiked with $1x10^{10}$ rEV by ODG. Next, inter-user variation was induced by sample replacement with PBS (see material and methods). The total number of particles was measured using NTA in scatter mode. The total number of rEV was measured using fNTA. From the latter the separtion efficiency was calculated (knowing that $1x10^{10}$ rEV were spiked) and implemented to normalize the number of scatter particles. Data is representative for figure 5 e.

Supplementary table 2

Healthy volunteers									
**Sample**	**scatter/mL**	**total scatter**	**recovery fNTA**	**total scatter normalized (fNTA)**	**particles/mL plasma (fNTA)**	**recovery ELISA**	**total scatter normalized (ELISA)**	**particles/mL plasma (ELISA)**	**absolute particles/mL**
HP1	1.79E+10	7.15E+10	34.88%	1.84E+11	6.72E+10	24.76%	2.89E+11	1.19E+11	3.58E+10
HP2	1.30E+10	5.18E+10	27.44%	1.89E+11	6.95E+10	26.19%	1.98E+11	7.40E+10	2.59E+10
HP3	2.26E+10	9.04E+10	29.84%	3.03E+11	1.26E+11	17.97%	5.03E+11	2.27E+11	4.52E+10
HP4	2.10E+10	8.40E+10	42.40%	1.98E+11	7.41E+10	48.48%	1.73E+11	6.16E+10	4.20E+10
HP5	1.67E+10	6.69E+10	28.96%	2.31E+11	9.05E+10	33.62%	1.99E+11	7.45E+10	3.34E+10
HP6	1.31E+10	5.23E+10	36.56%	1.43E+11	4.66E+10	35.35%	1.48E+11	4.90E+10	2.62E+10
HP7	9.66E+09	3.86E+10	28.40%	1.36E+11	4.30E+10	25.73%	1.50E+11	5.01E+10	1.93E+10
HP8	4.21E+09	1.68E+10	22.32%	7.54E+10	2.77E+10	17.29%	9.74E+10	3.87E+10	8.42E+09
HP9	8.40E+09	3.36E+10	28.84%	1.17E+11	4.83E+10	16.54%	2.03E+11	9.16E+10	1.68E+10
HP10	3.20E+09	1.28E+10	28.88%	4.43E+10	1.22E+10	32.85%	3.90E+10	9.48E+09	6.40E+09
HP11	6.10E+09	2.44E+10	26.24%	9.30E+10	3.65E+10	26.98%	9.04E+10	3.52E+10	1.22E+10
**Cancer patients**									
**Sample**	**scatter/mL**	**total scatter**	**recovery fNTA**	**total scatter normalized (fNTA)**	**particles/mL plasma (fNTA)**	**recovery ELISA**	**total scatter normalized (ELISA)**	**particles/mL plasma (ELISA)**	**absolute particles/mL**
CP1	1.05E+10	4.20E+10	19.12%	2.20E+11	8.48E+10	19.60%	2.14E+11	8.21E+10	2.10E+10
CP2	3.09E+10	1.24E+11	24.80%	4.99E+11	2.24E+11	23.11%	5.35E+11	2.43E+11	6.18E+10
CP3	1.26E+10	5.03E+10	26.48%	1.90E+11	7.00E+10	23.21%	2.17E+11	8.34E+10	2.52E+10
CP4	8.54E+09	3.42E+10	16.32%	2.09E+11	7.97E+10	18.48%	1.85E+11	6.74E+10	1.71E+10
CP5	3.26E+10	1.30E+11	23.44%	5.55E+11	2.53E+11	40.01%	3.25E+11	1.38E+11	6.51E+10
CP6	3.09E+10	1.23E+11	23.52%	5.25E+11	2.37E+11	60.11%	2.05E+11	7.76E+10	6.17E+10
CP7	1.41E+10	5.64E+10	21.50%	2.62E+11	1.21E+11	23.69%	2.38E+11	1.09E+11	2.82E+10
CP8	9.90E+09	3.96E+10	20.90%	1.89E+11	8.47E+10	24.96%	1.59E+11	6.93E+10	1.98E+10
CP9	2.53E+10	1.01E+11	22.50%	4.50E+11	2.15E+11	15.46%	6.55E+11	3.17E+11	5.06E+10
CP10	1.57E+10	6.26E+10	24.90%	2.51E+11	1.16E+11	17.30%	3.62E+11	1.71E+11	3.13E+10
CP11	1.44E+10	5.76E+10	24.40%	2.36E+11	1.08E+11	21.03%	2.74E+11	1.27E+11	2.88E+10
CP12	4.17E+10	1.67E+11	21.80%	7.64E+11	3.72E+11	27.17%	6.13E+11	2.97E+11	8.33E+10
CP13	1.32E+10	5.28E+10	17.60%	3.00E+11	1.40E+11	8.73%	6.05E+11	2.93E+11	2.64E+10
CP14	1.42E+09	5.66E+09	8.56%	6.61E+10	2.31E+10	4.28%	1.32E+11	5.61E+10	2.83E+09
CP15	3.72E+09	1.49E+10	11.30%	1.32E+11	5.58E+10	11.09%	1.34E+11	5.71E+10	7.44E+09
CP16	9.00E+09	3.60E+10	15.70%	2.29E+11	1.05E+11	11.36%	3.17E+11	1.48E+11	1.80E+10
CP17	1.03E+10	4.12E+10	13.80%	2.99E+11	1.39E+11	8.28%	4.98E+11	2.39E+11	2.06E+10
CP18	1.84E+10	7.34E+10	14.90%	4.93E+11	2.36E+11	15.66%	4.69E+11	2.25E+11	3.67E+10
CP19	7.32E+09	2.93E+10	12.36%	2.37E+11	1.08E+11	9.17%	3.19E+11	1.50E+11	1.46E+10
CP20	7.80E+09	3.12E+10	22.84%	1.37E+11	5.83E+10	14.96%	2.09E+11	9.43E+10	1.56E+10
CP21	3.82E+10	1.53E+11	33.44%	4.57E+11	2.04E+11	39.20%	3.90E+11	1.70E+11	7.65E+10
CP22	5.88E+10	2.35E+11	42.56%	5.53E+11	2.51E+11	32.46%	7.25E+11	3.37E+11	1.18E+11
CP23	2.28E+10	9.12E+10	22.64%	4.03E+11	1.76E+11	20.68%	4.41E+11	1.95E+11	4.56E+10
CP24	1.68E+10	6.71E+10	30.00%	2.24E+11	8.68E+10	22.28%	3.01E+11	1.26E+11	3.35E+10
CP25	3.46E+10	1.38E+11	36.56%	3.79E+11	1.64E+11	40.57%	3.41E+11	1.46E+11	6.92E+10
CP26	1.95E+10	7.79E+10	32.48%	2.40E+11	9.49E+10	29.04%	2.68E+11	1.09E+11	3.89E+10

Supplementary table 2: rEV accurately enumerate EV in patient samples. EV/mL plasma from healthy volunteers (n=11) and breast cancer patients (n=26) after separation by ODG, quantified with NTA and normalized by rEV quantification with fNTA and an ELISA for p24. Data is representative for figure 5 f.

Supplementary table 3

Sample	Dilution (times)	Gain	Shutter time (ms)
100 nm polystyrene beads	2,000	15	7.93
200 nm polystyrene beads	1,000	15	0.58
400 nm polystyrene beads	100	15	0.33
rEV	4,000	219	30.8
Urine EV	2,000	219	30.8
MCF7 EV	5,000	219	30.8
CAF EV	1,000	219	30.8
4T1 EV	10,000	219	30.8

Supplementary table 3: Camera settings for nanoparticle tracking analysis.
