# Supplementary material for: The generation and use of recombinant extracellular vesicles as biological reference material
Source: Nat Commun. 2019 Jul 23;10:3288. doi: 10.1038/s41467-019-11182-0 (PMC6650486; doi:10.1038/s41467-019-11182-0)
Supplement: Supplementary file 3 — Reporting Summary [file 41467_2019_11182_MOESM3_ESM.pdf]

nature research

Corresponding author(s): An Hendrix

Last updated by author(s): Jun 4, 2019

Reporting Summary

Nature Research wishes to improve the reproducibility of the work that we publish. This form provides structure for consistency and transparency in reporting. For further information on Nature Research policies, see Authors & Referees and the Editorial Policy Checklist.

Statistics

For all statistical analyses, confirm that the following items are present in the figure legend, table legend, main text, or Methods section.

n/a	Confirmed	
☐	☒	The exact sample size (*n*) for each experimental group/condition, given as a discrete number and unit of measurement
☐	☒	A statement on whether measurements were taken from distinct samples or whether the same sample was measured repeatedly
☐	☒	The statistical test(s) used AND whether they are one- or two-sided<br>*Only common tests should be described solely by name; describe more complex techniques in the Methods section.*
☐	☒	A description of all covariates tested
☒	☐	A description of any assumptions or corrections, such as tests of normality and adjustment for multiple comparisons
☐	☒	A full description of the statistical parameters including central tendency (e.g. means) or other basic estimates (e.g. regression coefficient) AND variation (e.g. standard deviation) or associated estimates of uncertainty (e.g. confidence intervals)
☒	☐	For null hypothesis testing, the test statistic (e.g. *F*, *t*, *r*) with confidence intervals, effect sizes, degrees of freedom and *P* value noted<br>*Give P values as exact values whenever suitable.*
☒	☐	For Bayesian analysis, information on the choice of priors and Markov chain Monte Carlo settings
☒	☐	For hierarchical and complex designs, identification of the appropriate level for tests and full reporting of outcomes
☒	☐	Estimates of effect sizes (e.g. Cohen's *d*, Pearson's *r*), indicating how they were calculated

Our web collection on statistics for biologists contains articles on many of the points above.

Software and code

Policy information about availability of computer code

Data collection	All software used for data collection is specified in the materials and methods section of each procedure.
Data analysis	All software used for data analysis is specified in the materials and methods section of each procedure.

For manuscripts utilizing custom algorithms or software that are central to the research but not yet described in published literature, software must be made available to editors/reviewers. We strongly encourage code deposition in a community repository (e.g. GitHub). See the Nature Research guidelines for submitting code & software for further information.

Data

Policy information about availability of data

All manuscripts must include a data availability statement. This statement should provide the following information, where applicable:

- Accession codes, unique identifiers, or web links for publicly available datasets
- A list of figures that have associated raw data
- A description of any restrictions on data availability

Proteomic data were submitted to PRIDE database (PXD010269). All relevant data of our experiments were submitted to the EV-TRACK knowledgebase (EV-TRACK ID: XP0801KW). The source data underlying figures 2, 3, 4, 5 and supplementary figures 1, 2, 5, 6, 7, 8, 10, 11 and 13 are provided as a source data file. All other relevant data that support the findings of this study are available from the corresponding author upon reasonable request.

Field-specific reporting

Please select the one below that is the best fit for your research. If you are not sure, read the appropriate sections before making your selection.

☒ Life sciences ☐ Behavioural & social sciences ☐ Ecological, evolutionary & environmental sciences

For a reference copy of the document with all sections, see nature.com/documents/nr-reporting-summary-flat.pdf

Life sciences study design

All studies must disclose on these points even when the disclosure is negative.

Sample size	The patient sample size for the proof of concept experiment (figure 5 f) was determined in relation to the practicable feasibility.
Data exclusions	No data was excluded from the analysis.
Replication	All experiments were validated in at least three independent experiments.
Randomization	No experimental groups were created in this study. Only a comparison was made between healthy indivuals and cancer patients.
Blinding	Blinding was not relevant to this study since only a comparison was made between healthy individuals and cancer patients

Reporting for specific materials, systems and methods

We require information from authors about some types of materials, experimental systems and methods used in many studies. Here, indicate whether each material, system or method listed is relevant to your study. If you are not sure if a list item applies to your research, read the appropriate section before selecting a response.

Materials & experimental systems

n/a	Involved in the study	
☐	☒	Antibodies
☐	☒	Eukaryotic cell lines
☒	☐	Palaeontology
☒	☐	Animals and other organisms
☐	☒	Human research participants
☒	☐	Clinical data

Methods

n/a	Involved in the study	
☒	☐	ChIP-seq
☐	☒	Flow cytometry
☒	☐	MRI-based neuroimaging

Antibodies

Antibodies used	The following primary and secondary antibodies were used for immunostaining: mouse monoclonal anti-ALIX (1:1000, #2171)and rabbit monoclonal anti-CD9 clone D3H4P (1:1000, #13403S) (Cell Signaling Technology, Danvers, MA, USA), mouse monoclonal anti-CD63 clone MEM-259 (1:200, #ab8219) and rabbit monoclonal anti-syntenin-1 (1:1000, #ab133267) Abcam, Cambridge, UK), mouse monoclonal anti-CD81 (1:1000, #SC-166029) and mouse monoclonal anti-TSG101 (1:100, #SC-7964) (Santa Cruz Biotechnology, Dallas, TX, USA), mouse monoclonal anti-flotillin-1 (1:1000, #610820) and rat anti-mouse CD9 (1:1000, #553758) (BD Biosciences, Franklin Lakes, NJ, USA), mouse monoclonal anti-green fluorescent protein (GFP) (1:1000, #MAB3580) (Merck Millipore, Billerica, MA, USA), , mouse monoclonal anti-α-tubulin (1:4000) (T5168, Sigma, Diegem, Belgium), sheep anti-mouse horseradish peroxidase-linked antibody (1:3000, #NA931V) and donkey anti-rabbit horseradish peroxidase-linked antibody (1:4000, #NA934V) (GE Healthcare Life Sciences, Uppsala, Sweden), . Immune electron microscopy was performed with a primary mouse monoclonal anti-CD63 antibody (1:50) (clone H5C6) (557305, BD Biosciences, Franklin Lakes, NJ, USA) and a rabbit anti-mouse IgG (1:2000) (Zymed Laboratories, San Francisco, CA, USA) to which 10 nm Gold-conjugated Protein A was added (1:70) (Cell Microscopy Core, University Medical Center Utrecht, The Netherlands). Antibodies used for immunoprecipitation were mouse monoclonal anti-CD81 antibody (MA5-13548, Thermo Fischer scientific, Erembodegem, Belgium), mouse monoclonal anti-CD63 (556019, BD Biosciences, Franklin Lakes, NJ, USA) and rabbit polyclonal anti-PEG (ab190652, Abcam, Cambridge, UK).
Validation	All antibodies were validated with appropriate positive and negative controls. Negative controls for immune precipitation experiments were included in the manuscript.

Eukaryotic cell lines

Policy information about cell lines

Cell line source(s)	Human HEK293T and MCF7 cells, mouse 4T1 cells were purchased from ATCC (Manassas, VA, USA). Human cancer associated

Cell line source(s)	fibroblasts were obtained as previously explained by De Wever et al.
Authentication	All cell lines, except mouse 4T1 cells, were authenticated.
Mycoplasma contamination	Cell cultures were regularly tested and confirmed negative for mycoplasma contamination using the MycoAlert Mycoplasma Detection Kit (Lonza, Verviers, Belgium).
Commonly misidentified lines (See ICLAC register)	Non commonly misidentified cell lines were used in this study.

Human research participants

Policy information about studies involving human research participants

Population characteristics	None covariate-relevant population characteristics were taken into account.
Recruitment	All participitants in this study were recruited and diagnosed by clinicians of the Ghent University Hospital.
Ethics oversight	Collection of patient samples was according to ethical committee of Ghent University Hospital approval and in accordance to relevant guidelines.

Note that full information on the approval of the study protocol must also be provided in the manuscript.

Flow Cytometry

Plots

Confirm that:

☒ The axis labels state the marker and fluorochrome used (e.g. CD4-FITC).

☒ The axis scales are clearly visible. Include numbers along axes only for bottom left plot of group (a 'group' is an analysis of identical markers).

☒ All plots are contour plots with outliers or pseudocolor plots.

☐ A numerical value for number of cells or percentage (with statistics) is provided.

Methodology

Sample preparation	rEV stocks were diluted in PBS and vortexed just before measurement. EV counts were determined by measuring each sample within an experiment for a fixed amount of time (30-120 s). The event rate was below 10,000/s to avoid coincident particle detection and occurrence of swarm.
Instrument	jet-in-air-based BD Influx flow cytometer (BD Biosciences, Franklin Lakes, NJ, USA) using an optimized configuration
Software	FlowJo software v 7.6.5 was used to analyse the flowcytomtry data in this study.
Cell population abundance	Cell population is not applicable in this study since only extracellular vesicles were measured.
Gating strategy	The gating strategy for extracellular vesicles was described previously by van der Vlist et al.

☒ Tick this box to confirm that a figure exemplifying the gating strategy is provided in the Supplementary Information.
